# Operative Versus Nonoperative Outcomes: A Cohort Study on Distal Biceps Tendon Rupture

**DOI:** 10.7759/cureus.93741

**Published:** 2025-10-02

**Authors:** Ahmad A Quzli, Hassan Jouni, Andrew P Dekker, Niyam Amanullah, Aiman Ishaq, Neil Ashwood

**Affiliations:** 1 Trauma and Orthopaedics, Wirral University Teaching Hospital NHS Foundation Trust, Wirral, GBR; 2 Otolaryngology/Ear, Nose, and Throat (ENT), Wirral University Teaching Hospital NHS Foundation Trust, Wirral, GBR; 3 Trauma and Orthopaedics, University Hospitals of Derby and Burton NHS Foundation Trust, Burton-on-Trent, GBR

**Keywords:** distal biceps rupture, distal biceps tendon, distal biceps tendon repair, nonoperative management, operative management, retrospective cohort, sports-related injuries, surgical complication

## Abstract

Background

Distal biceps tendon ruptures typically occur in middle-aged men following eccentric loading activities. While surgical repair is common, comparative data on operative versus nonoperative outcomes remain limited. We conducted a retrospective study to compare the outcomes of operative versus nonoperative management in Queen's Hospital Burton.

Methods

We reviewed the records of 72 patients (52 operative, 20 nonoperative) treated during the period of 2016-2023 for complete distal biceps tendon ruptures. All diagnoses were confirmed clinically and radiologically. Operative management was via a single anterior incision or a modified two-incision technique using cortical button fixation. Complications, range of motion, and return to activity were abstracted from clinical records documented at the time of care. Validated outcome measures, such as the Disabilities of the Arm, Shoulder, and Hand (DASH) and the Mayo Elbow Performance Score (MEPS), were not collected as part of routine care.

Results

Operative management was associated with a higher rate of complications, including nerve injuries and wound issues, though most patients ultimately regained a full range of motion and function. Nonoperative management resulted in minimal complications, with patients reporting return to their previous activity levels and only minor subjective strength deficits. Overall, operative repair offered greater strength recovery, particularly in supination, but at the cost of increased morbidity, whereas nonoperative management provided excellent functional outcomes with lower risk.

Conclusion

Nonoperative management appeared to be a reasonable option for selected patients in this cohort. Operative repair is effective but associated with a higher complication rate. Treatment should be individualised, balancing patient activity level, expectations, and risk profile.

## Introduction

Distal biceps tendon rupture is a relatively uncommon injury, accounting for approximately 3% of all tendon injuries, with an incidence of around 1.2 per 100,000 per year [1,2]. It most often affects middle-aged men engaged in heavy lifting or eccentric loading activities [3]. The biceps muscle plays an essential role in forearm supination and elbow flexion, and anatomical factors influence the supination moment arm [4,5]. Rupture of the distal tendon leads to decreased strength and endurance, particularly in supination. Surgical repair aims to restore function and strength but carries risks including posterior interosseous nerve injury, heterotopic ossification, and infection [6,7]. Several techniques have been described, including single- and double-incision approaches with various fixation methods [8-10]. Recent literature suggests that nonoperative treatment may provide satisfactory outcomes in selected patients, with lower complication rates and preserved function [11,12]. However, comparative evidence is limited by small cohorts, heterogeneity of techniques and follow-up, and inconsistent use of standardised outcome measures [11-13]. We therefore aimed to compare, within a single centre, operative and nonoperative management using clinical record-derived measures of complications, elbow range of motion, and return to work and activity from 2016 to 2023.

## Materials and methods

This retrospective study was conducted at Queen's Hospital Burton and included 72 patients diagnosed with complete distal biceps tendon rupture between January 2016 and December 2023. Diagnosis was confirmed by clinical examination and imaging (ultrasound or magnetic resonance imaging (MRI)). Patients were divided into two groups: operative (n=52) and nonoperative (n=20). Operative repair was performed using either a single anterior incision or a modified two-incision approach with cortical button fixation. Rehabilitation protocols varied between surgeons and physiotherapists. Postoperative care generally involved sling immobilisation for one to two weeks, mobilisation commencing between the first and second weeks, and progressive strengthening introduced between weeks 6 and 8 according to clinical progress. Nonoperative care began with mobilisation as pain allowed, with graded strengthening thereafter. Validated outcome measures, including the Disabilities of the Arm, Shoulder, and Hand (DASH) and the Mayo Elbow Performance Score (MEPS), were not part of routine care at our unit during 2016-2023 and were therefore unavailable for retrospective extraction. We did not attempt the retrospective collection of these measures to avoid recall bias and non-standardised timing. Accordingly, outcomes were abstracted from clinical records documented at the time of care, with elbow range of motion and complications documented by clinicians and return to work and activity recorded from patient self-report. Complications were defined as any adverse event recorded at follow-up, including transient nerve symptoms, wound-related issues (infection or granuloma), and reduced range of motion at the latest review. Patients could experience more than one complication, and percentages are reported per patient. Patients in both groups were assessed over a minimum follow-up period of six months.

## Results

Out of 72 patients, 52 underwent surgical repair, and 20 were managed nonoperatively. All patients were male, aged between 36 and 67 years (Table 1).

**Table 1:** Patient characteristics by treatment group

Characteristic	Operative (n=52)	Nonoperative (n=20)
Age, years (mean±SD)	47±9	50±12
Male sex n (%)	52 (100%)	20 (100%)
Dominant arm injured, n (%)	31 (60%)	10 (50%)
Follow-up, months (mean±SD)	12±6	12±6

In the operative group, complications were observed in 19 patients (36.5%), as tabulated in Table 2. These included posterior interosseous nerve palsy, radial artery injury, lateral cutaneous nerve numbness, granuloma, cubital tunnel syndrome, transient neurapraxia that subsequently resolved, wound infection, and reduced range of motion. No tendon re-rupture was observed. Despite the complication rate, the majority of patients regained a full range of motion and returned to baseline activity levels. Several patients developed transient nerve symptoms that resolved with conservative management.

**Table 2:** Outcomes by treatment group

Outcome/complication	Operative (n=52)	Nonoperative (n=20)
Any complication, n (%)	19 (36.5%)	0 (0%)
Posterior interosseous nerve palsy, n (%)	1 (1.9%)	0 (0%)
Arterial injury, n (%)	1 (1.9%)	0 (0%)
Wound infection, n (%)	1 (1.9%)	0 (0%)
Wound granuloma, n (%)	1 (1.9%)	0 (0%)
Re-rupture, n (%)	0 (0%)	0 (0%)
Lateral cutaneous nerve injury, n (%)	3 (5.8%)	0 (0%)
Reduced range of motion, n (%)	10 (19.2%)	0 (0%)
Cubital tunnel syndrome, n (%)	2 (3.8%)	0 (0%)
Transient neurapraxia, n (%)	7 (13.5%)	0 (0%)

In the nonoperative group, no complications were recorded. All patients reported a return to their pre-injury activity level for daily activities and work. Mild subjective loss of strength was noted in a few cases, particularly with supination, but did not impact daily function or return to work.

Overall, operative patients generally regained greater strength, especially in supination, but this benefit was offset by a higher complication burden. Nonoperative management was associated with less morbidity and high patient-reported functional satisfaction.

## Discussion

This retrospective cohort reveals that nonoperative management of distal biceps ruptures can result in excellent functional outcomes with minimal risk, whereas operative repair, though effective, is associated with some morbidity. In our series, 36.5% of operative patients experienced complications, higher than the 20-28% reported in meta-analyses and systematic reviews [7,13]. This difference likely reflects broader event capture and systematic recording at serial visits in our service, including transient neurapraxia and reduced range of motion at the latest review, and the influence of a multi-surgeon practice with variation in approach and rehabilitation.

Systematic reviews have summarised surgical complications, showing that while surgery typically restores supination and flexion strength, it also carries risks of neurological injury and heterotopic ossification [7,9]. Comparative studies report greater strength after repair, particularly for supination [3,11]. Validated outcome measures were not collected as part of routine care and were therefore unavailable for retrospective extraction, so strength comparisons were not evaluated, and the results focus on complications, range of motion, and return to activity. Biomechanical and fixation studies support cortical button fixation for robust repair strength; however, even with button fixation, neurological complications remain a concern [9,10].

The benefits of surgery appear most relevant to younger, high-demand patients whose occupations or recreational activities require robust supination strength. Nonoperative management, by contrast, spares patients' operative risk while often providing acceptable subjective function. Prior work has demonstrated that many conservatively treated patients adapt well with minimal impact on activities of daily living despite measurable strength deficits [11,12]. These observations are consistent with our nonoperative cohort, in which patients returned to baseline activity with only mild subjective loss of strength.

Recent large-scale analyses have examined complication profiles across techniques. Dunphy et al. analysed 784 surgical repairs and reported that while re-rupture is uncommon, neurological complications (including lateral antebrachial cutaneous nerve symptoms and occasional posterior interosseous nerve palsy) are not rare [14]. Kodde et al.'s systematic review comparing approaches and fixation methods found that double-incision techniques with bone tunnel fixation had fewer complications in some series, although heterogeneity limits firm conclusions [9]. Our series used anterior single-incision and modified two-incision repairs with cortical button fixation; the complication profile we observed is consistent with risks previously reported.

Rehabilitation strategy also influences outcomes. Studies suggest that early active range of motion protocols after repair can be safe and allow earlier restoration of function without increasing re-rupture risk [15,16]. In our cohort, rehabilitation protocols varied between surgeons and physiotherapists, which could influence recovery; therefore, differences in range of motion and return to activity cannot be attributed to treatment alone.

Overall, our results add to a growing body of evidence that many patients treated nonoperatively are satisfied and avoid the risks associated with surgery. A tailored approach that weighs functional demands, comorbidity, and personal preference, with shared decision-making, is recommended. High-quality prospective data with standardised outcome measures are needed to better define thresholds for operative treatment.

Limitations

This study has several limitations. Firstly, it was retrospective, non-randomised, and conducted at a single institution, which may introduce bias related to surgical technique and clinical documentation. Rehabilitation protocols were not standardised and varied between surgeons and physiotherapists, representing unmeasured post-treatment heterogeneity that may have influenced recovery of range of motion and return to work and activity and limits attribution of differences to treatment alone. Secondly, the sample size was relatively small, which limits statistical power and generalisability. Moreover, the cohort included only male patients, which restricts applicability to females and does not account for sex-based anatomical or functional differences [1]. Validated outcome measures (DASH, MEPS) were not collected as part of routine care and were therefore unavailable for retrospective extraction, which reduces comparability with studies that used these instruments [11,12]. Future work should prospectively collect standardised scoring with validated instruments, such as DASH and MEPS, at prespecified follow-up points to enhance comparability with published studies and facilitate inclusion in future syntheses.

## Conclusions

In this 72-patient retrospective cohort study, distal biceps tendon ruptures managed nonoperatively resulted in excellent function with no complications. Operative repair achieved comparable motion but was associated with a high complication rate. These findings suggest that nonoperative management appears to be a reasonable option for selected patients. Further prospective studies with larger samples and validated outcome measures are warranted to guide evidence-based practice.
